# Somatic Hypermutations Enhance Neutralization Breadth of *IGHV3‐53/3‐66* Public Antibodies Against SARS‐CoV‐2 Variants

**DOI:** 10.1002/iid3.70527

**Published:** 2026-09-16

**Authors:** Takeo Kuwata, Kyo Okazaki, Hiroshi Morioka, Yu Kaku, Mikiko Shimizu, Yoshimi Maruyama, Ahmed K. Hamdy, The Genotype to Phenotype Japan (G2P‐Japan) Consortium, Tateki Suzuki, Takao Hashiguchi, Kei Sato, Shuzo Matsushita

**Affiliations:** ^1^ Collaborative Research Program With the Chemo‐Sero‐Therapeutic Research Institute for Anti‐Viral Agents and Hematological Diseases, Joint Research Center for Human Retrovirus Infection Kumamoto University Kumamoto Japan; ^2^ Department of Analytical and Biophysical Chemistry, Faculty of Medical and Pharmaceutical Sciences Kumamoto University Kumamoto Japan; ^3^ Department of Clinical Infectious Diseases, Graduate School of Medical Sciences Nagoya City University Nagoya Japan; ^4^ Nagoya City University East Medical Center Nagoya Japan; ^5^ Laboratory of Medical Virology, Institute for Life and Medical Sciences Kyoto University Kyoto Japan; ^6^ Department of Microbiology and Immunology, Division of Systems Virology, The Institute of Medical Science The University of Tokyo Tokyo Japan

**Keywords:** neutralizing antibody, public antibody, SARS‐CoV‐2, variant

## Abstract

**Objective:**

Severe acute respiratory syndrome coronavirus 2 (SARS‐CoV‐2) continuously evolves to evade antibodies elicited by prior infection or vaccination. Most *IGHV3‐53/3‐66* public antibodies potently neutralize the prototype strain, but show limited activity against recent variants. However, some acquire broad neutralizing activity through accumulation of somatic hypermutations. We assessed whether non‐broadly neutralizing *IGHV3‐53/3‐66* antibodies could mature into broadly neutralizing antibodies.

**Methods:**

A series of mutant antibodies was constructed based on the *IGHV3‐53/3‐66* antibodies, 9‐105 and K4‐66. Neutralizing and binding activities were compared with the original antibodies.

**Results:**

Introducing six mutations frequently observed in broadly neutralizing antibodies markedly improved the neutralization and binding of 9‐105 against Omicron variants. Introducing Y66F into K4‐66 enhanced neutralization of variants including BA.4/5 and JN.1.

**Conclusion:**

These findings show that mutations within the *IGHV3‐53/3‐66* gene can enhance antibody breadth and potency, suggesting the potential of vaccine strategies to promote the maturation of these widely prevalent public antibodies.

Severe acute respiratory syndrome coronavirus 2 (SARS‐CoV‐2) variants continue to evolve, accumulating mutations in the receptor‐binding domain (RBD) of the Spike (S) protein, and thereby escaping from antibodies induced by prior infection or vaccination [1, 2, 3, 4, 5, 6]. Anti‐RBD antibodies encoded by the *IGHV3‐53/3‐66* gene are well known as public antibodies that are frequently induced in different individuals infected with early variants or vaccinated with prototype antigens [2, 3, 4, 5, 6]. Although most *IGHV3‐53/3‐66* public antibodies potently neutralize the prototype SARS‐CoV‐2 strain, they fail to neutralize recently circulating variants. This limitation is mainly attributed to immune imprinting, which restricts the antibody response to newly emerging variants [4]. Only a small number of *IGHV3‐53/3‐66* monoclonal antibodies (mAbs) have been reported to cross‐neutralize a wide range of variants [2, 3, 5, 6, 7]. These broadly neutralizing antibodies (bnAbs) harbor a higher number of somatic hypermutations (SHM) in the *IGHV3‐53/3‐66* gene than prototype‐specific mAbs, and several mutations are commonly shared among bnAbs. These observations suggest that prototype‐specific *IGHV3‐53/3‐66* public antibodies induced by early variant exposure may evolve into bnAbs capable of neutralizing recent variants through the acquisition of critical mutations. However, it remains unclear whether mutations commonly observed in bnAbs are sufficient to confer broadly neutralizing activity on non‐bnAb antibodies.

To examine the potential of non‐bnAb *IGHV3‐53/3‐66* public antibodies to mature into bnAbs, we generated a series of mutant antibodies based on mAb 9‐105 [1], which potently neutralizes pre‐Omicron variants, and evaluated their cross‐neutralizing activities against SARS‐CoV‐2 variants. Nine positions that frequently mutated in bnAbs were identified from two mAbs, K4‐66 and K1‐68, in the previous study [2] and 36 *IGHV3‐53/3‐66* mAbs in the CoV‐AbDab database [8], which neutralize XBB (Figure 1A). The most frequent bnAb‐associated mutations were Y66F (65.8% of 38 mAbs), F28I/L/V (63.2%), T29I (52.6%), S36R/A/N (52.6%), V55L/I/S (47.4%), S40T/N (44.7%), A96V/P/G (44.7%), N82D/S/T (36.8%), and S58P/A (26.3%) based on IMGT numbering (https://www.imgt.org). Among these, two mutations, F28I and V55L, are present in 9‐105. Six mutations, T29I, S36R, S40T, S58P, Y66F, and A96V, were introduced into 9‐105, generating five mutant antibodies (Figure 1B). The remaining N82D/S/T was not analyzed due to the low frequency in bnAbs and the location far from complementarity‐determining regions (CDR). Compared with the parental 9‐105, these mutant antibodies showed comparable neutralizing activity against the 614 G variant, but markedly enhanced activity against the BA.1, BA.2, and BA.4/5 variants (Figure 1C,D). The XBB.1.5 and XBB.1.16 variants were not neutralized by the parental 9‐105 or by mutants with partial mutations (9‐105M1–M4), but were neutralized by 9‐105M5, which contains all six mutations. None of the antibodies neutralized the JN.1 variant, although 9‐105M5 exhibited greater cross‐neutralizing activity than the other antibodies.

**FIGURE 1:**
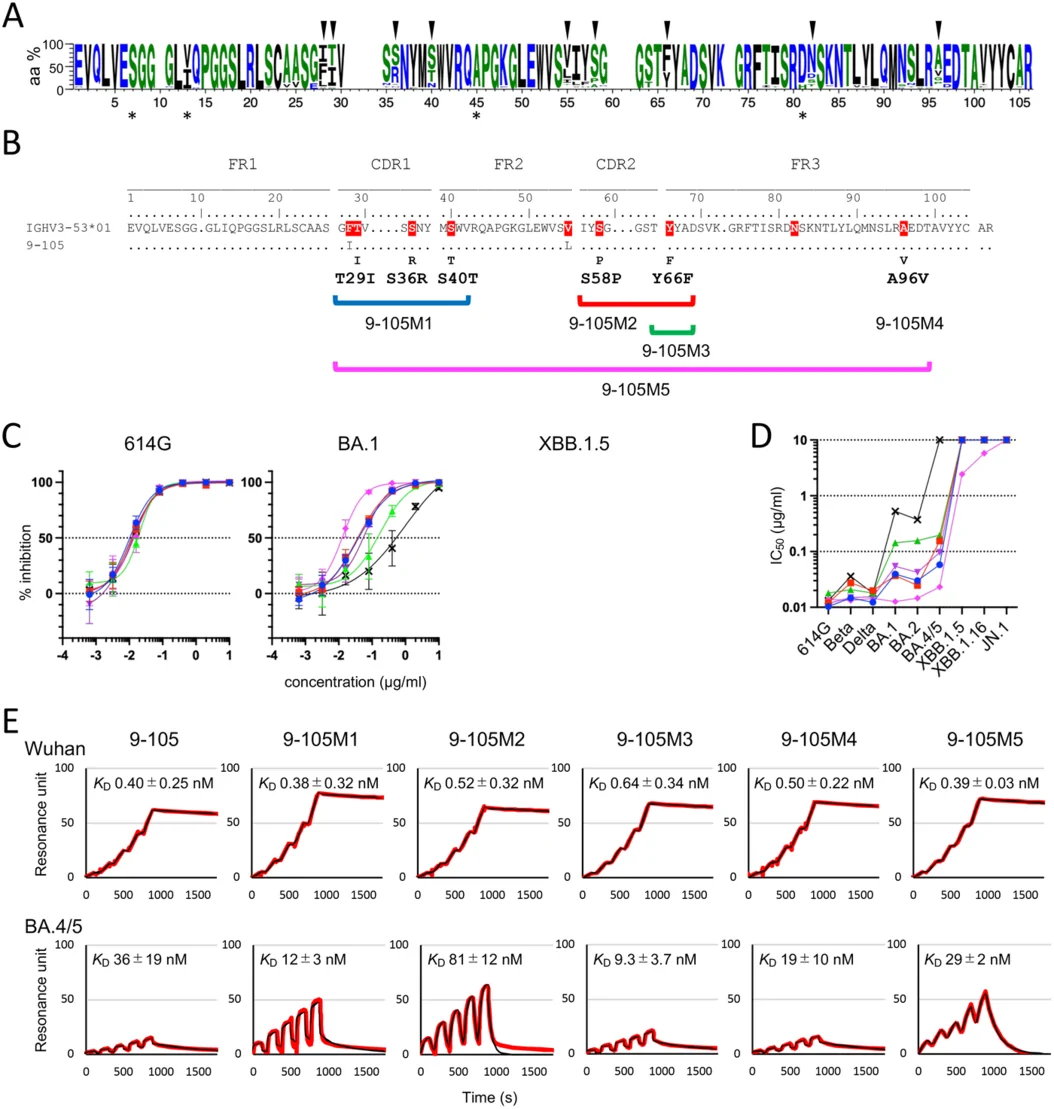
Enhanced neutralization breadth of *IGHV3‐53/3‐66* public antibody, 9‐105, by introducing bnAb‐associated mutations. (A) Amino acid frequencies in the heavy chains of 38 *IGHV3‐53/3‐66* bnAbs from the previous study [1] and CoV‐AbDab [8] are shown as sequence logos generated using WebLogo (http://weblogo.berkeley.edu). Nine positions associated with bnAb mutations are indicated by a triangle. Positions variable among *IGHV3‐53/3‐66* alleles are marked with an asterisk. IMGT numbering is used. (B) Amino acid sequences of 9‐105 and the germline gene IGHV3‐53*01 were aligned. The same amino acids were shown as dots. Nine positions of bnAb‐associated mutations are shown by red. Mutations introduced into mutant antibodies are indicated below the alignment. (C) Neutralizing activities of 9‐105 mutants against SARS‐CoV‐2 variants, 614G, BA.1, and XBB.1.5, are shown. Antibodies were produced in ExpiCHO cells and purified using protein A, as described [1, 2]. Neutralizing activity was determined by infection of 293FT ACE2/TMPRSS2 cells, as described [1, 2]. (D) Neutralizing potencies of 9‐105 mutants are summarized by IC_50_ (µg/mL) values against the 614G, Beta, Delta, BA.1, BA.2, BA.4/5, XBB.1.5, XBB.1.16, and JN.1 variants. Symbols and lines for 9‐105 mutants are same as those in Figure 1B. (E) Binding activities of 9‐105 mutants to Wuhan (upper) and BA.4/5 (lower) RBDs are shown. SPR analysis was performed by Biacore T200, as described [1]. All 9‐105 mutants were captured on the sensor chip using a Human Antibody Capture Kit at a capture level of 550–650 RU. A serially‐diluted Wuhan RBD (1–6 nM) or BA.4/5 RBD (2–8 nM [9‐105M5] and 10–50 nM [other mAbs]) was injected over immobilized mAbs. Dissociation constants (*K*_*D*_) are presented as mean ± standard deviation from three experiments.

Surface plasmon resonance (SPR) analysis revealed that binding to BA.4/5 RBD varied among the mutant antibodies, whereas binding to Wuhan RBD was similar across all antibodies, including the parental 9‐105 (Figure 1E). Notably, 9‐105M5 showed a markedly higher resonance unit (RU) than other antibodies, requiring a reduced concentration of BA.4/5 RBD for injection. A modest increase in RU was observed for 9‐105M1 and 9‐105M2, which carry mutations in CDR1 and CDR2 regions, respectively, whereas the RU values of 9‐105M3 and 9‐105M4 were comparable to that of 9‐105. The increased RU suggests enhanced binding of these mutant antibodies to BA.4/5 RBD compared with 9‐105. The binding affinity of 9‐105M3 to BA.4/5 RBD (9.3 nM) was stronger than those of the other antibodies (12–81 nM), suggesting that the Y66F substitution, the most frequent bnAb‐associated mutation, may contribute to increased affinity for BA.4/5 RBD.

To further examine the effect of bnAb‐associated mutations on neutralization breadth, the Y66F substitution was introduced into another *IGHV3‐53/3‐66* public antibody, K4‐66 [2] (Figure 2A). K4‐66 carries six bnAb‐associated mutations and, accordingly, neutralized all tested variants up to JN.1. However, it does not contain the Y66F substitution, the most frequently observed mutation among bnAbs (Figure 1A). The Y66F mutant of K4‐66 (K4‐66F) neutralized the tested variants more effectively than the parental K4‐66 (Figure 2B,C). In particular, neutralization potency against the Beta, BA.1, BA.4/5, and JN.1 variants was enhanced (2.6–3.4 fold). These results suggest that bnAb‐associated mutations can further enhance the potency of *IGHV3‐53/3‐66* public antibodies that already exhibit considerable breadth.

**FIGURE 2:**
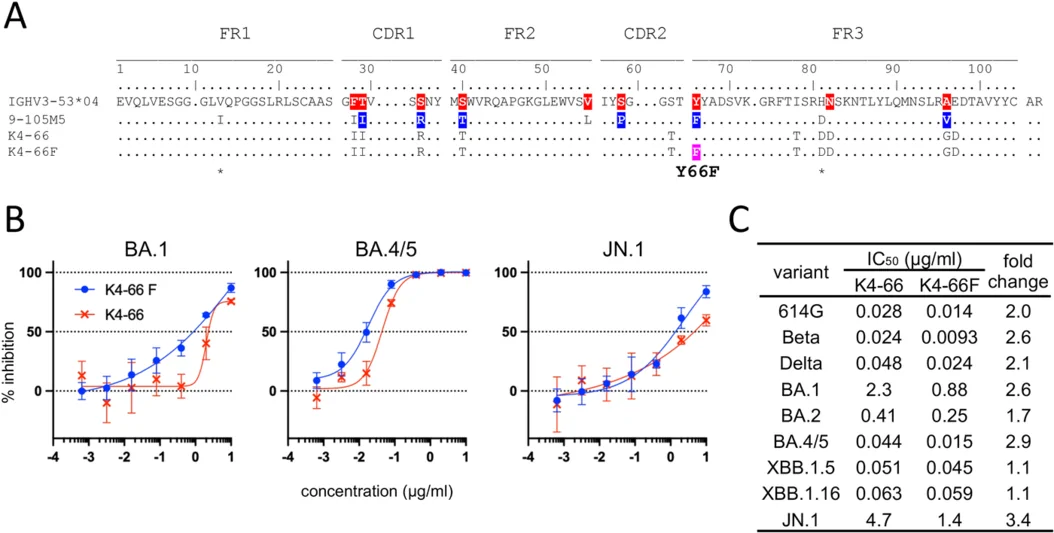
Enhanced neutralization potency of *IGHV3‐53/3‐66* public antibody, K4‐66, by introduction of the Y66F mutation. (A) Amino acid sequences of IGHV3‐53*04, 9‐105M5, K4‐66, and K4‐66F were aligned. The same amino acids were shown as dots. Nine positions of bnAb‐associated mutations are shown by red. The mutations introduced into 9‐105M5 are shown by blue. The Y66F mutation, which is introduced into K4‐66F, is shown by magenta. Two amino acid changes between IGHV3‐53*04 and IGHV3‐53*01 (Figure 1A) are indicated by asterisk. (B) Neutralizing activities of K4‐66 and K4‐66F against the BA.1, BA.4/5, and JN.1 variants are shown. (C) Neutralizing potencies of K4‐66 and K4‐66F are compared by IC_50_ (µg/mL) values against the 614G, Beta, Delta, BA.1, BA.2, BA.4/5, XBB.1.5, XBB.1.16, and JN.1 variants. Fold change was calculated by dividing the IC_50_ value of K4‐66 by that of K4‐66F.

Our study demonstrates that neutralization breadth and potency of *IGHV3‐53/3‐66* public antibodies can be enhanced by introducing mutations into the *IGHV3‐53/3‐66* gene. These findings suggest that prototype‐specific *IGHV3‐53/3‐66* public antibodies, induced by infection with early SARS‐CoV‐2 variants or vaccination with the prototype strain, have the potential to mature into bnAbs through accumulation of SHMs. Antibodies using the *IGHV1‐58* gene were also reported to mature into bnAbs [9]. The enhanced breadth of *IGHV3‐53/3‐66* public antibodies by SHMs was shown by introducing four mutations (G27E, T29I, S58P, and Y66F) to several *IGHV3‐53/3‐66* public antibodies [7]. The contribution of G27E was also reported by other investigators [10, 11], although the mutation frequency at G27 was low in our analysis (15.8%, Figure 1A). In addition, the importance of light chain gene usage was suggested [3]. Moreover, our results indicated that multiple mutations at critical positions in the *IGHV3‐53/3‐66* gene are required to achieve broad neutralization. Promoting the maturation of *IGHV3‐53/3‐66* public antibodies—already present in a large proportion of the population—may be one potential vaccine strategy to enhance protection against diverse SARS‐CoV‐2 variants.

## Author Contributions

**Takeo Kuwata:** writing – original draft, conceptualization, investigation, data curation, project administration. **Kyo Okazaki:** investigation. **Hiroshi Morioka:** investigation, validation, writing – review and editing. **Yu Kaku:** investigation, resources. **Mikiko Shimizu:** investigation, resources. **Yoshimi Maruyama:** investigation, resources. **Ahmed K. Hamdy:** resources. **Tateki Suzuki:** resources. **Takao Hashiguchi:** resources, funding acquisition, writing – review and editing. **Kei Sato:** resources. **Shuzo Matsushita:** supervision, funding acquisition, writing – review and editing.

## Ethics Statement

The authors have nothing to report.

## Consent

All authors have read and agreed to the published version of the manuscript.

## Conflicts of Interest

The authors declare no conflicts of interest.

## Supporting information


Supporting File

## Data Availability

The data that support the findings of this study are available from the corresponding author upon reasonable request.

## References

[1] Y. Kaku , T. Kuwata , H. M. Zahid , et al., “Resistance of SARS‐CoV‐2 Variants to Neutralization by Antibodies Induced in Convalescent Patients With COVID‐19,” Cell Reports 36, no. 2 (2021): 109385, 10.1016/j.celrep.2021.109385. 34237284 PMC8226103

[2] T. Kuwata , Y. Kaku , S. Biswas , et al., “Induction of *IGHV3‐53* Public Antibodies With Broadly Neutralising Activity Against SARS‐CoV‐2 Including Omicron Subvariants in a Delta Breakthrough Infection Case,” EBioMedicine 110 (2024): 105439, 10.1016/j.ebiom.2024.105439. 39488016 PMC11565539

[3] J. Dingus , D. K. Yoo , S. Kumar , et al., “Affinity Maturation and Light‐Chain‐Mediated Paratope Diversification Anticipates Viral Evolution,” Cell Reports 45, no. 1 (2026): 116800, 10.1016/j.celrep.2025.116800. 41474620 PMC12952975

[4] X. Niu , F. Jian , Y. Li , et al., “IGHV3‐53 Antibody Abundance Drives Divergent SARS‐CoV‐2 Immune Imprinting,” bioRxiv. Preprint Posted Online December 24 (2025): 2025.12.18.694353, 10.64898/2025.12.18.694353.

[5] L. Li , X. Chen , Z. Wang , et al., “Breakthrough Infection Elicits Hypermutated IGHV3‐53/3‐66 Public Antibodies With Broad and Potent Neutralizing Activity Against SARS‐CoV‐2 Variants Including the Emerging EG.5 Lineages,” PLoS Pathogens 19, no. 12 (2023): e1011856, 10.1371/journal.ppat.1011856. 38048356 PMC10721163

[6] F. Jian , A. Z. Wec , L. Feng , et al., “Viral Evolution Prediction Identifies Broadly Neutralizing Antibodies to Existing and Prospective SARS‐CoV‐2 Variants,” Nature Microbiology 10, no. 8 (2025): 2003–2017, 10.1038/s41564-025-02030-7. PMC12313522 40494884

[7] H. Lv , Z. Feng , Q. W. Teo , et al., “Somatic Evolution of a Cross‐Reactive Germline Antibody That Expands Its Breadth to Neutralize New SARS‐CoV‐2 Variants,” bioRxiv. Preprint Posted Online March 25 (2026): 2026.03.20.713312, 10.64898/2026.03.20.713312.

[8] M. I. J. Raybould , A. Kovaltsuk , C. Marks , and C. M. Deane , “CoV‐AbDab: The Coronavirus Antibody Database,” Bioinformatics 37, no. 5 (2021): 734–735, 10.1093/bioinformatics/btaa739. 32805021 PMC7558925

[9] M. V. Stankov , M. Bruhn , M. Hoffmann , et al., “JN.1‐Adapted Vaccination Is Associated With Readjustment of Ancestral Memory B Cells Toward Neutralization Within the JN.1 Antigenic Space,” Nature Communications 17, no. 1 (2026): 7266, 10.1038/s41467-026-76035-z. PMC13400639 42498786

[10] M. Bruhn , M. Obara , A. Salam , et al., “Diversification of the VH3‐53 Immunoglobulin Gene Segment by Somatic Hypermutation Results in Neutralization of SARS‐CoV‐2 Virus Variants,” European Journal of Immunology 54, no. 7 (2024): 2451056, 10.1002/eji.202451056. 38593351

[11] Q. Yan , X. Huang , B. Liu , et al., “AI Recognizes Convergent Somatic Hypermutation Signatures to Allow the Discovery of Variant‐Resilient Broadly Neutralizing Antibodies,” bioRxiv. Preprint Posted Online December 12 (2025): 2025.12.10.693372, 10.64898/2025.12.10.693372.

