# Supplementary material for: Somatic Hypermutations Enhance Neutralization Breadth of IGHV3‐53/3‐66 Public Antibodies Against SARS‐CoV‐2 Variants
Source: Immun Inflamm Dis. 2026 Sep 16;14(9):e70527. doi: 10.1002/iid3.70527 (PMC13580150; doi:10.1002/iid3.70527)
Supplement: Supplementary file 1 [file IID3-14-e70527-s001.pdf]

Immunology

Letter to the Editor

Somatic hypermutations enhance neutralization breadth of IGHV3-53/3-66 public antibodies against SARS-CoV-2 variants

Submission ID 1481179a-e7dc-4c23-85d7-fc5f47259993

Submission Version Initial Submission

PDF Generation 21 May 2026 20:03:04 EST by Atypon ReX

Authors

Dr. Takeo Kuwata
Corresponding Author
Submitting Author

ORCiD
https://orcid.org/0000-0001-7793-6585

Affiliations
- Collaborative Research Program with the Chemo-Sero-Therapeutic Research Institute for Anti-viral Agents and Hematological Diseases, Joint Research Center for Human Retrovirus infection, Kumamoto University, Kumamoto, Japan

Dr. Kyo Okazaki

Affiliations
- Department of Analytical and Biophysical Chemistry, Faculty of Medical and Pharmaceutical Sciences, Kumamoto University, Kumamoto, Japan

Dr. Hiroshi Morioka

Affiliations
- Department of Analytical and Biophysical Chemistry, Faculty of Medical and Pharmaceutical Sciences, Kumamoto University, Kumamoto, Japan

Dr. Yu Kaku

Affiliations
- Department of Clinical Infectious Diseases, Graduate School of Medical Sciences, Nagoya City University, Aichi, Japan
- Nagoya City University East Medical Center, Aichi, Japan

Mrs. Mikiko Shimizu

Affiliations

- Collaborative Research Program with the Chemo-Sero-Therapeutic Research Institute for Anti-viral Agents and Hematological Diseases, Joint Research Center for Human Retrovirus infection, Kumamoto University, Kumamoto, Japan

Mrs. Yoshimi Maruyama

Affiliations

- Collaborative Research Program with the Chemo-Sero-Therapeutic Research Institute for Anti-viral Agents and Hematological Diseases, Joint Research Center for Human Retrovirus infection, Kumamoto University, Kumamoto, Japan

Dr. Ahmed Khairy Hamdy

ORCiD
https://orcid.org/0000-0002-1830-9132

Affiliations

- Collaborative Research Program with the Chemo-Sero-Therapeutic Research Institute for Anti-viral Agents and Hematological Diseases, Joint Research Center for Human Retrovirus infection, Kumamoto University, Kumamoto, Japan

Dr. Tateki Suzuki

Affiliations

- Labolatory of Medical Virology, Institute for Life and Medical Sciences, Kyoto University, Kyoto, Japan

Dr. Takao Hashiguchi

Affiliations

- Labolatory of Medical Virology, Institute for Life and Medical Sciences, Kyoto University, Kyoto, Japan

Dr. Kei Sato

Affiliations

- Division of Systems Virology, Department of Microbiology and Immunology, The Institute of Medical Science, The University of Tokyo, Tokyo, Japan

Dr. Shuzo Matsushita

Affiliations

- Collaborative Research Program with the Chemo-Sero-Therapeutic Research Institute for Anti-viral Agents and Hematological Diseases, Joint Research Center for Human Retrovirus infection, Kumamoto University, Kumamoto, Japan

Files for peer review

All files submitted by the author for peer review are listed below. Files that could not be converted to PDF are indicated; reviewers are able to access them online.

Name	Type of File	Size	Page
VH353improve.docx	Main Document - MS Word	49.4 KB	Page 4
figure.pptx	Figure	5.4 MB	Page 13

Somatic hypermutations enhance neutralization breadth of *IGHV3-53/3-66* public antibodies against SARS-CoV-2 variants

Takeo Kuwata[1,*], Kyo Okazaki[2], Hiroshi Morioka[2], Yu Kaku[3, 4], Mikiko Shimizu[1], Yoshimi Maruyama[1], Ahmed Khairy Hamdy[1], The Genotype to Phenotype Japan (G2P-Japan) Consortium, Tateki Suzuki[5], Takao Hashiguchi[5], Kei Sato[6] and Shuzo Matsushita[1,*]

[1]Collaborative Research Program with the Chemo-Sero-Therapeutic Research Institute for Anti-viral Agents and Hematological Diseases, Joint Research Center for Human Retrovirus infection, Kumamoto University, Kumamoto, Japan

[2]Department of Analytical and Biophysical Chemistry, Faculty of Medical and Pharmaceutical Sciences, Kumamoto University, Kumamoto, Japan

[3]Department of Clinical Infectious Diseases, Graduate School of Medical Sciences, Nagoya City University, Aichi, Japan

[4]Nagoya City University East Medical Center, Aichi, Japan

[5]Labolatory of Medical Virology, Institute for Life and Medical Sciences, Kyoto University, Kyoto, Japan

[6]Division of Systems Virology, Department of Microbiology and Immunology, The Institute of Medical Science, The University of Tokyo, Tokyo, Japan

*Correspondence to: Takeo Kuwata (tkuwata@kumamoto-u.ac.jp) or Shuzo Matsushita (shuzo@kumamoto-u.ac.jp)

Joint Research Center for Human Retrovirus infection, Kumamoto University

5-1 Oe Honmachi, Chuo-ku, Kumamoto 862-0973, Japan

Funding

This study was supported by grants from the Japan Agency for Medical Research and Development (JP23ym0126048, JP25jf0126002 and JP223fa627009) and Japan Society for Promotion of Science (Core-to-Core Program).

Keywords

SARS-CoV-2, neutralizing antibody, variant, public antibody

Text

To the Editor,

Severe acute respiratory syndrome coronavirus 2 (SARS-CoV-2) variants continue to evolve, accumulating mutations in the receptor binding domain (RBD) of the Spike (S) protein, and thereby escaping from antibodies induced by prior infection or vaccination[1,2,3,4,5,6]. Anti-RBD antibodies encoded by the *IGHV3-53/3-66* gene are well known as public antibodies that are frequently induced in different individuals infected with early variants or vaccinated with prototype antigens[2,3,4,5,6]. Although most *IGHV3-53/3-66* public antibodies potently neutralize the prototype SARS-CoV-2 strain, they fail to neutralize recently circulating variants. This limitation is mainly attributed to immune imprinting, which restricts the antibody response to newly emerging variants[4]. Only a small number of *IGHV3-53/3-66* monoclonal antibodies (mAbs) have been reported to cross-neutralize a wide range of variants[2,3,5,6,7]. These broadly neutralizing antibodies (bnAb) harbor a higher number of somatic hypermutations (SHM) in the *IGHV3-53/3-66* gene than prototype-specific mAbs, and several mutations are commonly shared among bnAbs. These observations suggest that prototype-specific *IGHV3-53/3-66* public antibodies induced by early variant exposure may evolve into bnAbs capable of neutralizing recent variants through the acquisition of critical mutations. However, it

remains unclear whether mutations commonly observed in bnAbs are sufficient to confer broadly neutralizing activity on non-bnAb antibodies.

To examine the potential of non-bnAb *IGHV3-53/3-66* public antibodies to mature into bnAbs, we generated a series of mutant antibodies based on mAb 9-105[1], which potently neutralizes pre-Omicron variants, and evaluated their cross-neutralizing activities against SARS-CoV-2 variants. Eleven positions that frequently mutated in bnAbs were identified from 39 *IGHV3-53/3-66* mAbs in the CoV-AbDab database that neutralize XBB (Fig. 1A)[8]. The most frequent bnAb-associated mutations were Y66F (64.1% of 39 mAbs), F28I/L/V (61.5%), T29I (51.3%), S36R/A/N (51.3%), S40T/N (43.6%), A96V/P/G (43.6%), V55L/I/S (41.0%), N82D/S/T (35.9%), S58P/A (25.6%), A25V (17.5%) and S64T/N (17.5%) based on IMGT numbering (https://www.imgt.org). Among these, T29I, S36R, S40T, S58P, Y66F and A96V mutations were introduced into 9-105, generating five mutant antibodies (Fig. 1A). Compared with the parental 9-105, these mutant antibodies showed comparable neutralizing activity against the 614G variant, but markedly enhanced activity against the BA.1, BA.2 and BA.4/5 variants (Fig. 1B and C). The XBB.1.5 and XBB.1.16 variants were not neutralized by the parental 9-105 or by mutants with partial mutations (9-105M1~M4), but were neutralized by 9-105M5, which contains all six mutations. None of the antibodies neutralized the JN.1 variant, although

9-105M5 exhibited greater cross-neutralizing activity than the other antibodies.

Surface plasmon resonance (SPR) analysis revealed that binding to BA.4/5 RBD varied among the mutant antibodies, whereas binding to Wuhan RBD was similar across all antibodies, including the parental 9-105 (Fig. 1D). Notably, 9-105M5 showed a markedly higher resonance unit (RU) than other antibodies, requiring a reduced concentration of BA.4/5 RBD for injection. A modest increase in RU was observed for 9-105M1 and 9-105M2, which carry mutations in the complementarity-determining region 1 (CDR1) and CDR2 regions, respectively, whereas the RU values of 9-105M3 and 9-105M4 were comparable to that of 9-105. The increased RU suggests enhanced binding of these mutant antibodies to BA.4/5 RBD compared with 9-105. The binding affinity of 9-105M3 to BA.4/5 RBD (9.3 nM) was stronger than those of the other antibodies (12~81 nM), suggesting that the Y66F substitution, the most frequent bnAb-associated mutation, may contribute to increased affinity for BA.4/5 RBD.

To further examine the effect of bnAb-associated mutations on neutralization breadth, the Y66F substitution was introduced into another *IGHV3-53/3-66* public antibody, K4-66[2] (Fig. 2A). K4-66 carries six bnAb-associated mutations and, accordingly, neutralized all tested variants up to JN.1. However, it does not contain the Y66F substitution, the most frequently observed mutation among bnAbs. The Y66F mutant of K4-66 (K4-66F)

neutralized the tested variants more effectively than the parental K4-66 (Fig. 2B and C). In particular, neutralization potency against the Beta, BA.1, BA.4/5 and JN.1 variants was enhanced (2.6~3.4 fold). These results suggest that bnAb-associated mutations can further enhance the potency of *IGHV3-53/3-66* public antibodies that already exhibit considerable breadth.

Our study demonstrates that neutralization breadth and potency of *IGHV3-53/3-66* public antibodies can be enhanced by introducing mutations into the *IGHV3-53/3-66* gene. These findings suggest that prototype-specific *IGHV3-53/3-66* public antibodies, induced by infection with early SARS-CoV-2 variants or vaccination with the prototype strain, have the potential to mature into bnAbs through accumulation of SHMs. The enhanced breadth by SHMs was also shown by introducing five mutations (G27E, T29I, S58P and Y58F) to several *IGHV3-53/3-66* public antibodies[7]. In addition, the importance of light chain gene usage was suggested[3]. Moreover, our results indicated that multiple mutations at critical positions in the *IGHV3-53/3-66* gene are required to achieve broad neutralization. Promoting the maturation of *IGHV3-53/3-66* public antibodies - already present in a large proportion of the population - may represent an effective vaccine strategy for protect ion against diverse SARS-CoV-2 variants.

Author Contributions

Takeo Kuwata, and Shuzo Matsushita designed this study. Takeo Kuwata performed neutralization assay and wrote the manuscript. Kyo Okazaki and Hiroshi Morioka performed SPR analyses. Takeo KUWATA, Yu Kaku, Mikiko Shimizu, Yoshimi Maruyama and Ahmed Khairy Hamdy prepared recombinant mAbs. Takao Hashiguchi prepared RBD proteins. Kei Sato constructed plasmids to express the S protein. All authors accessed and verified the underlying data, and read and approved the final manuscript.

Acknowledgements

We thank CoV-AbDab for the useful database of antibodies against SARS-CoV-2. This research is the cooperative research project with The Chemo-Sero-Therapeutic Research Institute.

Ethics Statement

The authors have nothing to report.

Consent

All authors have read and agreed to the published version of the manuscript.

Conflicts of Interest

The authors declare no conflicts of interest.

Data Availability Statement

All the data will be available upon reasonable request to the corresponding author.

References

1. Kaku Y, Kuwata T, Zahid HM, et al. Resistance of SARS-CoV-2 variants to neutralization by antibodies induced in convalescent patients with COVID-19. *Cell Rep*. 2021;36(2):109385. doi:10.1016/j.celrep.2021.109385

2. Kuwata T, Kaku Y, Biswas S, et al. Induction of *IGHV3-53* public antibodies with broadly neutralising activity against SARS-CoV-2 including Omicron subvariants in a Delta breakthrough infection case. *eBioMedicine*. 2024;110:105439. doi:10.1016/j.ebiom.2024.105439

3. Dingus J, Yoo DK, Kumar S, et al. Affinity maturation and light-chain-mediated paratope diversification anticipates viral evolution. *Cell Reports*. 2026;45(1). doi:10.1016/j.celrep.2025.116800

4. Niu X, Jian F, Li Y, et al. IGHV3-53 antibody abundance drives divergent SARS-CoV-2 immune imprinting. *bioRxiv*. Preprint posted online December 24, 2025:2025.12.18.694353. doi:10.64898/2025.12.18.694353

5. Li L, Chen X, Wang Z, et al. Breakthrough infection elicits hypermutated IGHV3-53/3-66 public antibodies with broad and potent neutralizing activity against SARS-CoV-2 variants including the emerging EG.5 lineages. *PLOS Pathogens*. 2023;19(12):e1011856. doi:10.1371/journal.ppat.1011856

6. Jian F, Wec AZ, Feng L, et al. Viral evolution prediction identifies broadly neutralizing antibodies to existing and prospective SARS-CoV-2 variants. *Nat Microbiol*. 2025;10(8):2003-2017. doi:10.1038/s41564-025-02030-7

7. Lv H, Feng Z, Teo QW, et al. Somatic evolution of a cross-reactive germline antibody that expands its breadth to neutralize new SARS-CoV-2 variants. *bioRxiv*. Preprint posted online March 25, 2026:2026.03.20.713312. doi:10.64898/2026.03.20.713312

8. Raybould MIJ, Kovaltsuk A, Marks C, Deane CM. CoV-AbDab: the coronavirus antibody database. *Bioinformatics*. 2021;37(5):734-735. doi:10.1093/bioinformatics/btaa739

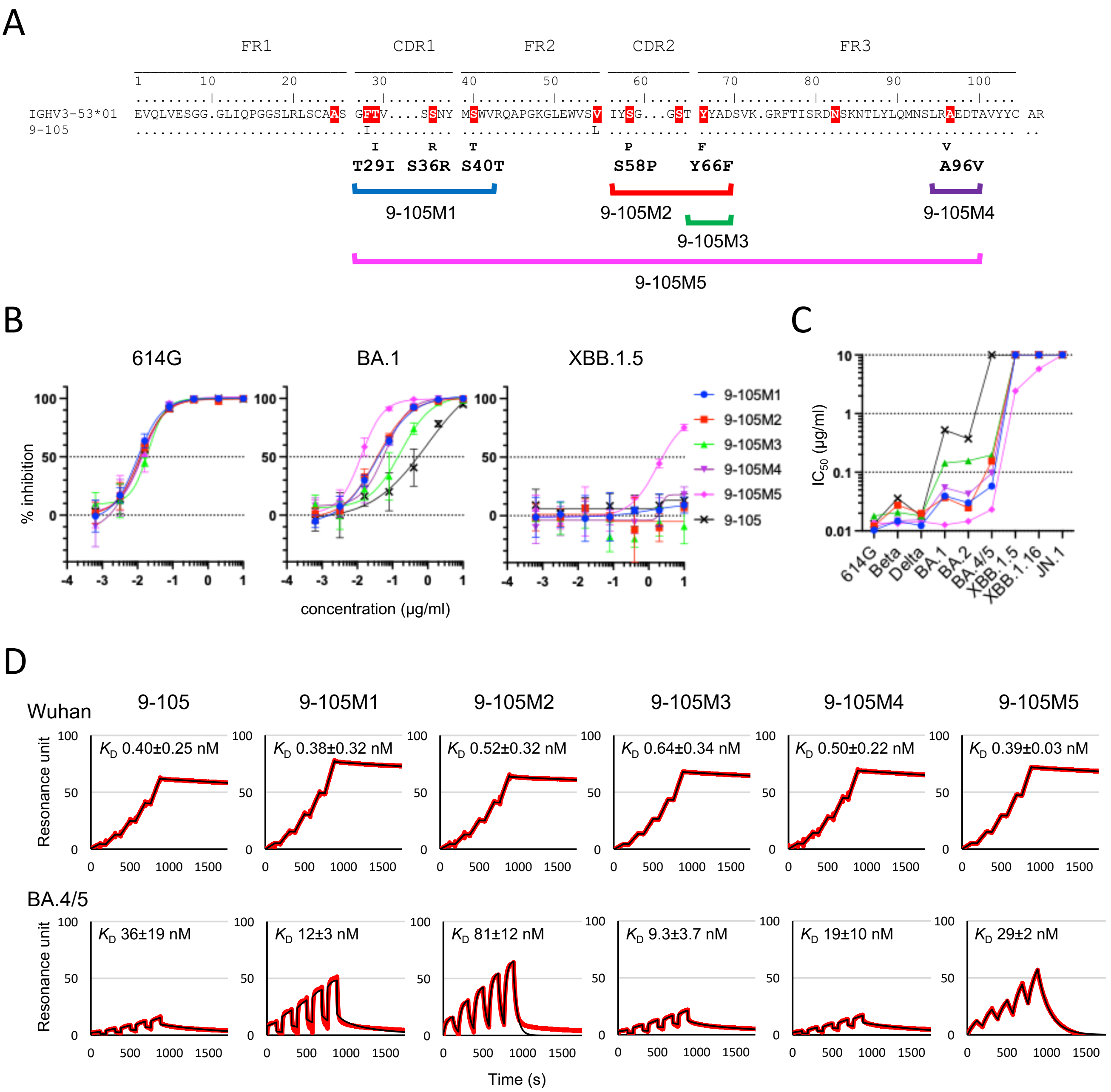

Fig.1. Enhanced neutralization breadth of *IGHV3-53/3-66* public antibody, 9-105, by introducing bnAb-associated mutations. (A) Amino acid sequences of 9-105 and the germline gene *IGHV3-53*01* were aligned. The same amino acids were shown as dots. Eleven positions of bnAb-associated mutations are shown by red. Mutations introduced into mutant antibodies are indicated below the alignment. (B) Neutralizing activities of 9-105 mutants against SARS-CoV-2 variants, 614G, BA.1 and XBB.1.5, are shown. Neutralizing activity was determined by infection of 293FT ACE2/TMPRSS2 cells, as described[1,2]. (C) Neutralizing potencies of 9-105 mutants are summarized by IC_{50} (μg/ml) values against the 614G, Beta, Delta, BA.1, BA.2, BA.4/5, XBB.1.5, XBB.1.16 and JN.1 variants. Symbols and lines for 9-105 mutants are same as those in Fig. 1B. (D) Binding activities of 9-105 mutants to Wuhan (upper) and BA.4/5 (lower) RBDs are shown. SPR analysis was performed by Biacore T200 using Human IgG capture kit, as described[1]. A serially-diluted Wuhan RBD (1-6 nM) or BA.4/5 RBD (2-8 nM [9-105M5] and 10-50 nM [other mAbs]) was injected over immobilized mAbs. Dissociation constants (K_D) are presented as mean ± standard deviation from three experiments.

A

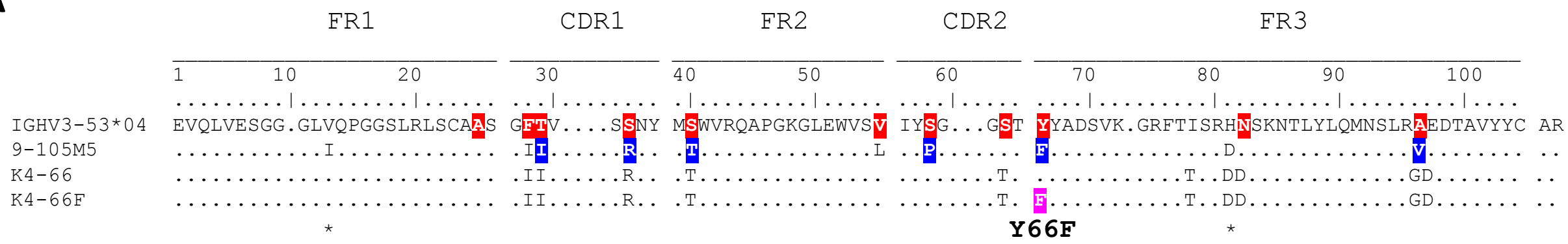

B

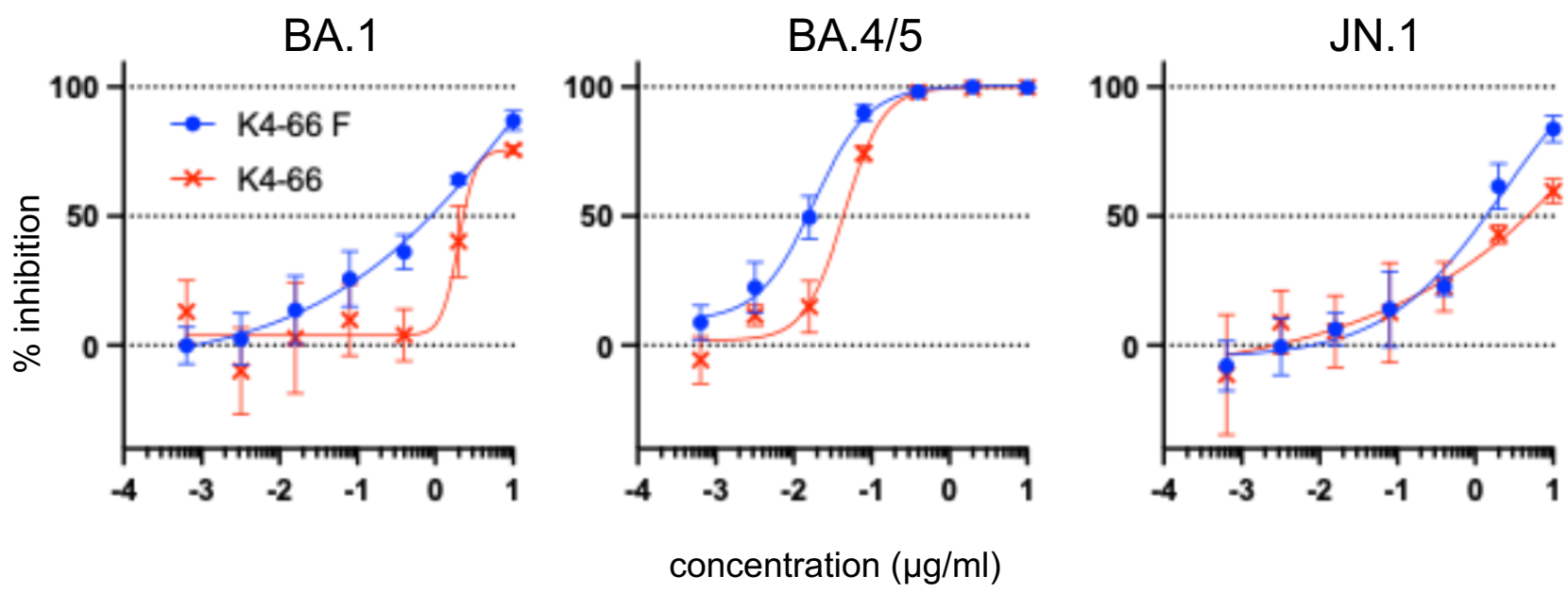

C

variant	IC_{50} (µg/ml) K4-66	IC_{50} (µg/ml) K4-66F	fold change
614G	0.028	0.014	2.0
Beta	0.024	0.0093	2.6
Delta	0.048	0.024	2.1
BA.1	2.3	0.88	2.6
BA.2	0.41	0.25	1.7
BA.4/5	0.044	0.015	2.9
XBB.1.5	0.051	0.045	1.1
XBB.1.16	0.063	0.059	1.1
JN.1	4.7	1.4	3.4

Fig.2. Enhanced neutralization potency of *IGHV3-53/3-66* public antibody, K4-66, by introduction of the Y66F mutation. (A) Amino acid sequences of *IGHV3-53*04*, 9-105M5, K4-66 and K4-66F were aligned. The same amino acids were shown as dots. Eleven positions of bnAb-associated mutations are shown by red. The mutations introduced into 9-105M5 are shown by blue. The Y66F mutation, which is introduced into K4-66F, is shown by magenta. Two amino acid changes between *IGHV3-53*04* and *IGHV3-53*01* (Fig. 1A) are indicated by asterisk. (B) Neutralizing activities of K4-66 and K4-66F against the BA.1, BA.4/5 and JN.1 variants are shown. (C) Neutralizing potencies of K4-66 and K4-66F are compared by IC_{50} (µg/ml) values against the 614G, Beta, Delta, BA.1, BA.2, BA.4/5, XBB.1.5, XBB.1.16 and JN.1 variants. Fold change was calculated by dividing the IC_{50} value of K4-66 by that of K4-66F.
